# Characterization of Mixed-Species Biofilm Formed by *Vibrio parahaemolyticus* and *Listeria monocytogenes*

**DOI:** 10.3389/fmicb.2019.02543

**Published:** 2019-11-08

**Authors:** Ping Chen, Jing Jing Wang, Bin Hong, Ling Tan, Jun Yan, Zhaohuan Zhang, Haiquan Liu, Yingjie Pan, Yong Zhao

**Affiliations:** ^1^College of Food Science and Technology, Shanghai Ocean University, Shanghai, China; ^2^Shanghai Engineering Research Center of Aquatic Product Processing and Preservation, Shanghai, China; ^3^Laboratory of Quality and Safety Risk Assessment for Aquatic Products on Storage and Preservation (Shanghai), Ministry of Agriculture and Rural Affairs, Shanghai, China; ^4^Engineering Research Center of Food Thermal-Processing Technology, Shanghai Ocean University, Shanghai, China

**Keywords:** *Vibrio parahaemolyticus*, *Listeria monocytogenes*, foodborne pathogen, mixed-species biofilms, minimum biofilm inhibitory concentration

## Abstract

Mixed-species biofilms are the predominant form of biofilms found in nature. Research on biofilms have typically concentrated on single species biofilms and this study expands the horizon of biofilm research, where the characterization and dynamic changes of mono and mixed-species biofilms formed by the pathogens, *Vibrio parahaemolyticus* and *Listeria monocytogenes* were investigated. Compared to mono-species biofilm, the biomass, bio-volume, and thickness of mixed-species biofilms were significantly lower, which were confirmed using crystal violet staining, confocal laser scanning microscopy and scanning electron microscopy. Further experimental analysis showed these variations might result from the reduction of bacterial numbers, the down-regulation of biofilm-regulated genes and loss of metabolic activity in mixed-species biofilm. In addition, *V. parahaemolyticus* was located primarily on the surface layers of the mixed-species biofilms thus accruing competitive advantage. This competitive advantage was evidenced in a higher *V. parahaemolyticus* population density in the mixed-species biofilms. The adhesion to surfaces of the mixed-species biofilms were also reduced due to lower concentrations of extracellular polysaccharide and protein when the structure of the mixed-species was examined using Raman spectral analysis, phenol-sulfuric acid method and Lowry method. Furthermore, the minimum biofilm inhibitory concentration to antibiotics obviously decreased when *V. parahaemolyticus* co-exited with *L. monocytogenes*. This study firstly elucidated the interactive behavior in biofilm development of two foodborne pathogens, and future studies for biofilm control and antibiotic therapy should take into account interactions in mixed-species biofilms.

## Introduction

More than 80% of bacteria exist in hydrated extracellular polymeric substance (EPS) called biofilms, which allows them to survive in adverse environments (Costerton et al., 1999; Hall-Stoodley et al., 2004). Pathogenic biofilms provide a source for contaminating food (Carpentier and Cerf, 2010) and these biofilms can be resistant to sanitizers (Elexson et al., 2014). It is estimated that billions of dollars are spent every year worldwide to deal with food contamination, equipment damage, and human diseases from microbial biofilms (Yang et al., 2011). Therefore, elucidating the mechanism of biofilm formation of bacteria will facilitate the development of effective strategies for removing bacterial biofilms in clinical, industrial, and agricultural environments.

*Vibrio parahaemolyticus* and *Listeria monocytogenes* are foodborne pathogens commonly found in seafood, aquatic products, ready-to-eat or raw foods and water (Kathariou, 2002; Su and Liu, 2007; Norhana et al., 2010; Grace et al., 2011). *V. parahaemolyticus* infection in human beings often leads to acute gastroenteritis with nausea, headache and low grade fever (Su and Liu, 2007; World Health Organization [WHO], 2012). *L. monocytogenes* infection may cause listeriosis (de Grandi et al., 2018; Radoshevich and Cossart, 2018) and the mortality rate is high where almost 1/3 infected cases will be fatal (Grace et al., 2011). *V. parahaemolyticus* and *L. monocytogenes* can coexist in different seafood products and in post-harvest processing sites and these are potential sources of cross contamination (Liao et al., 2015; Zhang et al., 2015a; Niu et al., 2018).

*Vibrio parahaemolyticus* and *L. monocytogenes* have been reported to easily form biofilms during food processing and storage (Han et al., 2016; Pang et al., 2019). These studies have explored biofilm formation of *V. parahaemolyticus* or *L. monocytogenes* based on a mono-species model (Blackman and Frank, 1996; Djordjevic et al., 2002; Borucki et al., 2003; Shime-Hattori et al., 2006; Song et al., 2017; Ahmed et al., 2018; Chen et al., 2018). Although studies for simultaneous detection of *V. parahaemolyticus* and *L. monocytogenes* in aquatic products have been reported previously (Zarei et al., 2012; Lee et al., 2014; Zhang et al., 2015b; Cho et al., 2016), no studies on mixed-species biofilms formation of *V. parahaemolyticus* and *L. monocytogenes* exist. More importantly, the behavior of microorganisms in a mixed biofilm differs from those in a mono-species biofilm (Parijs and Steenackers, 2018), especially resistance to antimicrobials. Therefore, the effects of interactions between different microorganisms during the formation and establishment of mixed biofilms are urgently needed (Ren et al., 2014).

The aim of this study was to firstly establish an *in vitro* experimental model for characterizing dynamic changes in mono- and mixed-species biofilms formed by *V. parahaemolyticus* and *L. monocytogenes*. This *in vitro* model will then be used for mechanistic understanding of biofilm adhesion, architectural structure, biofilm-regulated genes expression, metabolic activity, EPS formation, spatial localization and differences in antibiotic resistance of mono- and mixed-species biofilms.

## Materials and Methods

### Bacterial Strains and Culture Preparation

Four Strains were used to establish mono- and mixed-species biofilm model. Two standard strains of *V. parahaemolyticus* ATCC17802 (VP802), *L. monocytogenes* ATCC19115 (LM115) were purchased from the American Type Culture Collection and two isolates of *V. parahaemolyticus* VP24 (GenBank ID: MN536754), *L. monocytogenes* LM5 (GenBank ID: MN536755) were simultaneously isolated from the same frozen salmon that purchased from local supermarket. These strains were individually stored in tryptic soy broth (TSB, Beijing Land Bridge Technology Company, Ltd., Beijing, China) mixed with 25% glycerol at −80°C. *V. parahaemolyticus* was streaked onto thiosulfate-citrate-bile salts-sucrose (TCBS, Beijing Land Bridge Technology Company, Ltd., Beijing, China) agar plates and incubated at 37°C for 12 h. Individual colony was inoculated into 9 mL TSB supplemented with 2.5% NaCl and incubated overnight at 37°C with shaking at 180 rpm. *L. monocytogenes* was streaked onto PALCAM (PALCAM; Beijing Land Bridge Technology Company, Ltd., Beijing, China) plates with selective additives and incubated at 37°C for 30 h. Individual colony was grown in 9 mL brain heart infusion (BHI, Beijing Land Bridge Technology Company, Ltd., Beijing, China) and incubated overnight at 37°C with shaking at 180 rpm. The bacterial concentrations were diluted to ∼8 Log CFU mL^–1^ for use (Niu et al., 2018).

### Mono- and Mixed-Species Biofilm Formation

Biofilm formation experiments were performed as described previously with minor modification (Burmolle et al., 2006; Chen et al., 2015). The bacteria cultures and the TSB medium were transferred into a 24-well polystyrene microtiter plate at a ratio of 1:100 to develop the biofilm. The experimental groups were listed as follows: (1) 10 μL VP802 + 990 μL TSB; (2) 5 μL VP802 + 5 μL LM115 + 990 μL TSB; (3) 10 μL LM115 + 990 μL TSB; (4) 10 μL VP 24 + 990 μL TSB; (5) 5 μL VP24 + 5 μL LM5 + 990 μL TSB; (6) 10 μL LM5 + 990 μL TSB; (7) only 1 mL TSB was selected as control. Detailed culture temperatures and times are specified in each specific method.

### Quantification of Biofilm by Crystal Violet Staining (CV)

The CV assay was applied to evaluate biofilm formation as previously described (Antoniani et al., 2010; Han et al., 2017). Mono- and mixed-species biofilms were incubated at 25°C in 24-well microplate for 12, 24, 36, 48, 60, and 72 h with six independent experiments each time. Afterward, the planktonic cells were removed and the biofilm was washed two times with 0.1M phosphate-buffered saline (PBS). The formed biofilms were fixed at 55°C for 10 min and then stained with 1 mL of 0.1% (w/v) crystal violet (Sangon Biotech, Co., Ltd., Shanghai, China) for 15 min, rinsed three times with 1 mL of 0.1M PBS, and dissolved in 1 mL of 95% ethanol for 30 min. Two hundred microliter of the solution was transferred into 96-well microplate, and the optical density was measured at 600 nm by BioTek Synergy 2 (Winooski, VT, United States).

### Biofilm Imaging by Confocal Laser Scanning Microscopy (CLSM)

The confocal laser scanning microscopy (CLSM) was applied to visualize the biofilm as previously described (Tan et al., 2018). Mono- and mixed-species biofilms were formed on round glass slide (diameter is 14 mm) in 24-well microplate at 25°C for 12, 24, 36, 48, 60, and 72 h. After incubation, biofilms were washed twice with 1 mL 0.1M PBS to remove planktonic cells. Then, these were fixed with 4% (w/v) glutaraldehyde for 30 min at 4°C, followed by rinsing twice with 0.1M PBS. The fixed biofilms were stained with SYBR Green I (Sangon Biotech, Co., Ltd., Shanghai, China) for 30 min at room temperature, after which the excess fluorescent dye was removed and air dried. The experiment process needs to be protected from light. All images were captured with a Zeiss LSM 710 confocal laser scanning microscope (LSM710, Carl Zeiss AG, Germany). The 20 × objective was used to monitor SYBR Green I fluorescence excited at 488 nm and emitted at 500–550 nm. For each sample, the representative images of five separate sites on the glass slide were acquired randomly. Structural parameters of biofilm architecture were extracted from three-dimensional CLSM images by the ISA-2 software, a software package developed by the Biofilm Structure-Function research group, Center for Biofilm Engineering, Montana State University for the purpose of quantifying biofilm structures (Beyenal et al., 2004).

### Visualization of the Biofilms Using Scanning Electron Microscopy (SEM)

After 36 h incubation, mono- and mixed-species biofilms were formed on round slide (diameter is 14 mm) in 24-well microplate at 25°C. Mature biofilms were fixed overnight with 2.5% glutaraldehyde at 4°C. Afterward, the biofilms were washed twice with 0.1M PBS and dehydrated sequentially with ethanol (30, 50, 70, 80, 90, and 100% [twice]; 10 min each). Samples were coated with 5 nm Nano-gold in Turbomolecular pumped Sputter coater (Q150T ES PLUS, United Kingdom) and observed by Extreme-resolution Analytical Field Emission scanning electron microscope (SEM) (Tescan Mira 3 MH, Czechia). All SEM images were magnified by 20,000 times. The images were acquired for three independent replicate.

### Enumeration of Biofilm and Planktonic Cells by qPCR

The biofilm and planktonic cells were quantified by quantitative PCR (qPCR) according to the previous method with slight modification (Ren et al., 2014; Luk et al., 2018). Mono- and mixed-species mature biofilms were cultured at 25°C for 36 h. And then 1 mL planktonic cells were transferred into 1.5 mL tubes. The biofilm cells were suspended by vortexing and scraping with 1 mL 0.1M PBS and transferred into 1.5 mL tubes (Jackson et al., 2001). The bacterial cells were collected by centrifugation at 10,000 rpm for 1 min.

Total DNA was extracted using the TIANamp Bacteria DNA Kit (Tiangen Biotech Beijing, Co., Ltd., China) according to the manufacturer’s instruction. The qPCR was performed to quantify the biofilm and planktonic cells of *V. parahaemolyticus* and *L. monocytogenes* in mature biofilms. Primers used for detecting *V. parahaemolyticus* and *L. monocytogenes* are listed in Table 1. To construct standard curves, standard plasmids were prepared with the pLB Vector System (Tiangen Biotech Beijing, Co., Ltd., China) using thermolabile hemolysin gene (*tlh*) PCR products from a pure culture of *V. parahaemolyticus* and hemolysin gene (*hly*A) PCR products from a pure culture of *L. monocytogenes*. The exact concentration of plasmid was measured with a Quant-iT^TM^ PicoGreen^®^ dsDNA Reagent and Kits (Invitrogen, Thermo Fisher Scientific, United States). The standard plasmids were 10-fold serially diluted by DNase/RNase-free ddH_2_O from 10^8^ to 10^2^ copies/mL.

**TABLE 1:** Nucleotide sequence of primers for target bacteria.

**Target bacterium**	**Target gene**		**Nucleotide sequence** **(5′→3′)**	**PCR product size (bp)**	**References**
	***tlh***	***hly*A**			
*Vibrio parahaemolyticus*	+	−	F: ACTCAACACAAGAAGAGATCGACAA R: GATGAGCGGTTGATGTCCAA	208	Nordstrom et al., 2007; Zhang et al., 2015b
*Listeria monocytogenes*	−	+	F: ACTTCGGCGCAATCAGTGA R: TTGCAACTGCTCTTTAGTAACAGCTT	137	Omiccioli et al., 2009; Zhang et al., 2015b

*“±” indicates positive/negative real-time qPCR signal.*

Absolute qPCR was performed in a final volume of 20 μL: 10 μL FastStart Universal SYBR Green Master (Rox) (Roche, Co., Switzerland), 0.6 μL forward and reverse primers, 2 μL template DNA and 6.8 μL DNase/RNase-free ddH_2_O. PCR was conducted in ABI 7500 Fast real-time PCR system (Applied Biosystems, Foster City, CA, United States) with the following profiles: 95°C for 2 min, followed by 35 cycles of denaturation at 95°C for 15 s, and annealing at 60°C for 60 s. The results analysis was performed by 7500 Software v2.0.6.

### Transcriptional Analysis

Total RNA was extracted from biofilm cells using Bacteria RNA Extraction Kit (Vazyme Biotech, Co., Ltd., China) according to the manufacturer’s instruction, and quantified by BioTek Synergy 2 (Waltham, MA, United States). Complementary DNA (cDNA) was synthesized through random hexamer primed reactions using a HiScript III RT SuperMix for qPCR (+gDNA wiper) (Vazyme Biotech, Co., Ltd., China). Afterward, RT-qPCR was performed in a final volume of 20 μL: 10 μL 2õ ChamQ Universal SYBR qPCR Master Mix (Vazyme Biotech, Co., Ltd., China), 1 μL forward and reverse primers, 2 μL template cDNA and 6 μL DNase/RNase-free ddH_2_O. Primers used for RT-qPCR were shown in Supplementary Table S1. RT-qPCR reactions were carried out in a 7500 Fast Real-Time PCR system (Applied Biosystems, Waltham, MA, United States) with the following profiles: 95°C for 2 min, followed by 35 cycles of denaturation at 95°C for 15 s, and annealing at 60°C for 60 s. Each PCR reaction was conducted in triplicate, and controls without template were included. Then the results calculated by normalizing target genes to respective reference gene based on the 2^–ΔΔ*Ct*^ method.

### Evaluation of Cell Metabolic Activity of Biofilm by XTT Assay

XTT reduction assay was performed to assess the metabolic activity of biofilm as previously described with minor modification. XTT (Sangon Biotech, Co., Ltd., Shanghai, China) was dissolved in 0.1M PBS at a final concentration of 1 mg/mL, and then filter-sterilized with 0.22 μm membrane. Menadione solution (0.4 mM; Sangon Biotech, Co., Ltd., Shanghai, China) was prepared in acetone immediately before each assay. For each assay, XTT solution was mixed with menadione solution at a volume ratio of 20:1.

Mono- and mixed-species biofilms were incubated at 25°C in 24-well microplates for 36 h with six independent experiments. The biofilms were washed twice with 1 mL of 0.1M PBS to remove planktonic cells. Next, 790 μL of 0.1M PBS, 200 μL of 1 mg/mL XTT and 10 μL of 0.4 mM menadione solution were transferred into each well of 24-well microplates. The plates were incubated in dark at 37°C for 3 h. The colorimetric changes were measured at 492 nm by BioTek Synergy 2.

### Fluorescence *in situ* Hybridization (FISH)

Biofilm samples were pre-treated using the method described previously with slight modification (Karygianni et al., 2014; Liu et al., 2017). The mature biofilms were fixed with 4% (w/v) paraformaldehyde for 30 min, followed by washing twice with 1 mL 0.1M PBS (pH = 7.2). Each specimen was incubated with 100 μL lysozyme solution for 10 min at 37°C, and washed twice with 1 mL 0.1M PBS. To reduce background fluorescence, the specimens were treated with 1% H_2_O_2_ in methanol for 30 min at 55°C and then rinsed twice with 1 mL 0.1M PBS.

Fluorescence *in situ* hybridization (FISH) was carried out using DNA bacteria universal FISH kit (Guangzhou Exons Biological Technology, Co., Ltd., China). Oligonucleotide probes are commercially available and listed in Table 2. Probes for 16S rRNA of *V. parahaemolyticus* and *L. monocytogenes* were labeled with sulfoindocyanine dye indocarbocyanine (Cy3) and aminomethylcoumarin (AMCA), respectively. The maximum excitation wavelengths for Cy3 and AMCA were 554 and 350 nm. The maximum emission wavelengths for Cy3 and AMCA were 568 and 450 nm. Biofilm images were acquired by CLSM with 63 × oil-immersion objective and their bio-volume were quantified by ISA-2 software depending on 15 image stacks.

**TABLE 2:** Specific oligonucleotide probes for target bacteria in FISH.

**Target bacterium**	**Target gene**	**Probe sequence (5′→3′)**	**References**
*Vibrio parahaemolyticus*	16S rRNA	5′-CY3-ACTTTGTGAGATTCGCTCCACCTCG-CY3-3′	Sawabe et al., 2009
*Listeria monocytogenes*	16S rRNA	5′-AMCA-ACCTCGCGGCTTCGCGAC-AMCA-3′	Wagner et al., 1998; Schmid et al., 2010

### Extracellular Polymeric Substance (EPS) Analysis

Extracellular polymeric substance (EPS) of mono- and mixed-species biofilms formed by VP802 and LM115 were extracted by probe sonication extraction method (Liu et al., 2007; Han et al., 2017). The optical density of each suspended culture was initially measured at OD_595 *nm*_. Subsequently, planktonic cells were discarded. And then the mature biofilm was washed twice with 0.1M PBS and collected by vortexing and scraping in 1 mL 0.01M KCl solution. Afterward, the biofilm cells were treated by a sonicator (VCX 500, SONICS, Newtown, CT, United States) for four cycles with 5 s operation and 5 s pause at a power level of 60 w. After sonication, samples were centrifuged for 20 min (3000 rpm, 4°C), and the supernatant was collected and filtered through a 0.22 μm membrane filter for future EPS analysis (Sangon Biotech, Co., Ltd., Shanghai, China).

Raman Spectroscopy of EPS was conducted by a Senterra R200-L Dispersive Raman Microscope (Bruker Optics, Ettlingen, Germany). The Raman measurements were recorded with an accumulation time of 60 s in the 425–1300 cm^–1^ range equipped with a diode laser at 785 nm and 50 × objective with a laser power of 3 mW. Raman spectrum was determined as the average of six measurements at different sites on the EPS and analyzed using the Bruker OPUS software.

The amount of carbohydrate and protein were quantified by the phenol-sulfuric acid method and Lowry method (Nakamura et al., 2013; Janissen et al., 2015) and expressed as OD_490 *nm*_/OD_595 *nm*_ and OD_750 *nm*_/OD_595 *nm*_, respectively.

### Biofilm Antimicrobial Susceptibility Test

Minimum biofilm inhibitory concentration (MBIC) of antibiotics (ciprofloxacin and cefotaxime sodium) were determined for the mono- and mixed-species biofilms after 36 h incubation by the method described previously (Pierce et al., 2008; Harrison et al., 2010; Mathias et al., 2010). Biofilms were grown on peg lids in 96-well microtiter plates at 25°C, and rinsed twice by submersing the peg lid into the wells of the microtiter plate containing the 0.1M PBS. A 100 μl aliquot of the antimicrobial solution was serially diluted in TSB. The concentration of tested antibiotics ranged from 0 to 128 μg/mL. Then, the peg lid with biofilm was placed into the TSB with antibiotics and incubated for 24 h at 25°C. MBIC is defined as the lowest concentration at which there were no detectable bacteria after 24 h incubation at 25°C.

### Statistical Analysis

All experiments were tested at least in triplicate. The values were expressed as the mean ± standard deviation. Statistical analysis was performed using SPSS statistical package 24.0 (SPSS, Inc., Chicago, IL, United States). Differences at *p-*value < 0.05 were considered statistically significant. The figures were processed by Origin pro 9.0 (Origin Lab, Corp., Northampton, MA, United States).

## Results

### Development and Structural Characteristics of Mono- and Mixed-Species Biofilms

**FIGURE 1:**
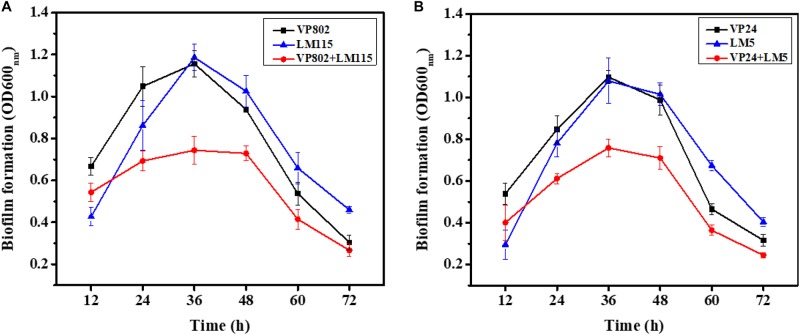
Mono- and mixed-species biofilms formation after 12, 24, 36, 48, 60, and 72 h incubation using CV assay. **(A)** Mono-, mixed-species biofilms formed by *Vibrio parahaemolyticus* VP802 and *Listeria monocytogenes* LM115; **(B)** mono-, mixed-species biofilm formed by *V. parahaemolyticus* VP24 and *L. monocytogenes* LM5. The black square, blue triangle represent mono-species biofilm formed by *V. parahaemolyticus* and *L. monocytogenes*, respectively. The red circle represents mixed-species biofilms formed by *V. parahaemolyticus* and *L. monocytogenes*. Error bars represent the standard deviation of triplicate experiments.

To explore the biofilm-forming capacity of *V. parahaemolyticus* and *L. monocytogenes*, a CV assay was used to detect the dynamics of the development of mono- and mixed-species biofilms after 12, 24, 36, 48, 60, and 72 h incubation (Figure 1). After a 12 h incubation, mono species biofilm formation of *V. parahaemolyticus* (VP802: OD_600 *nm*_ = 0.67 ± 0.04; VP24: OD_600 *nm*_ = 0.54 ± 0.05) was higher than that *L. monocytogenes* (LM115: OD_600 *nm*_ = 0.43 ± 0.04; LM5: OD_600 *nm*_ = 0.30 ± 0.08). With the increase of incubation time from 24 to 48 h, these single biofilms grew rapidly and reached maturation stage. Biofilm formation reached a maximum at 36 h and there was no significant difference in the OD_600 *nm*_ values of *L. monocytogenes* and *V. parahaemolyticus*. The maximum biofilm formation of VP802, VP802 + LM115, LM115, VP24, VP24 + LM5, and LM5 were 1.16 ± 0.06, 0.74 ± 0.07, 1.19 ± 0.06, 1.10 ± 0.03, 0.76 ± 0.04, and 1.08 ± 0.11, respectively. After 60 and 72 h incubation, the biofilm of all strains was reduced, suggesting that the biofilm was disintegrating. In contrast, biofilms formed by co-cultivation of two foodborne pathogenic strains were reduced relative to mono-species biofilms (*p* < 0.05) after 24 to 60 h incubation.

**FIGURE 2:**
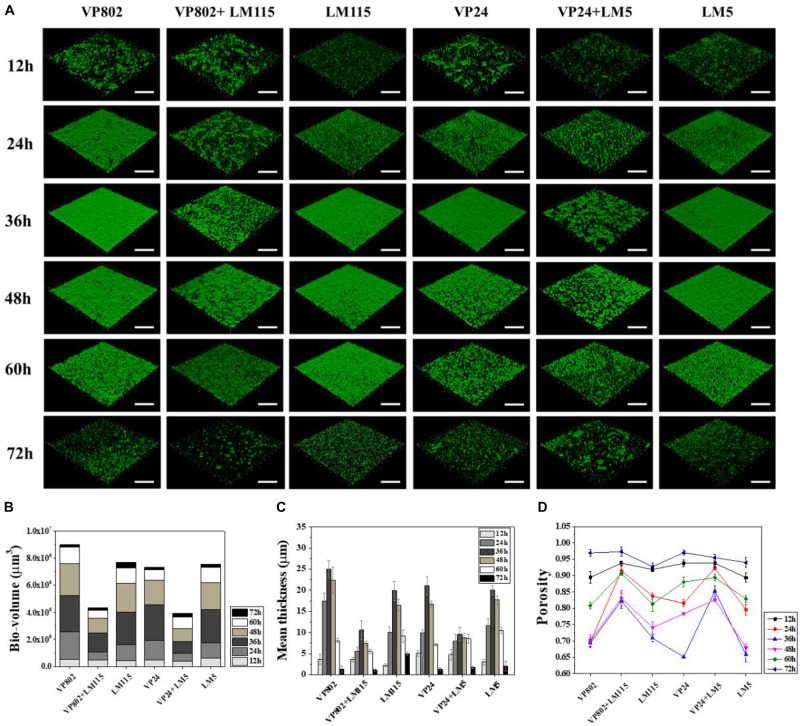
Biofilm developments of single- and mixed- species monitored by CLSM. **(A)** CLSM images (captured under the 20õ objective) showing the mono- and mixed-species biofilms formed by *V. parahaemolyticus* (VP802, VP24) and *L. monocytogenes* (LM115, LM5) after 12, 24, 36, 48, 60, and 72 h cultivation, respectively. Scale bar: 50 μm. Quantification of structural parameters in biofilm based on the CLSM images using ISA2 software: **(B)** Bio-volume, **(C)** Biofilm mean thickness, **(D)** Biofilm porosity. Error bars represent standard deviation of three biological replicates.

**FIGURE 3:**
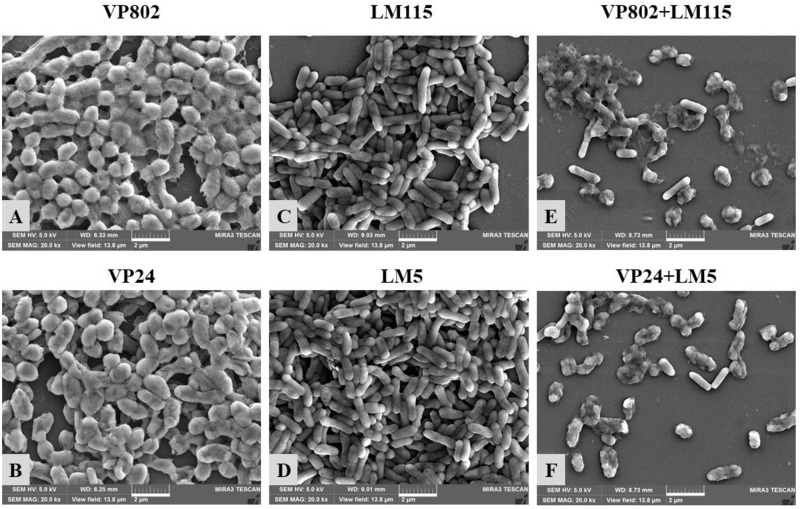
Scanning electron microscopy (SEM) images of mono- and mixed-species biofilms formed by *V. parahaemolyticus* and *L. monocytogenes*. Scale bar represented 2 μm. Pictures were representative of three independent experiments with three replicates each. **(A)** *V. parahaemolyticus* VP802; **(B)** *V. parahaemolyticus* VP24; **(C)** *L. monocytogenes* LM115; **(D)** *L. monocytogenes* LM5; **(E)** Mixed-species VP802+LM115; **(F)** Mixed-species VP24+LM5.

Confocal laser scanning microscopy was further employed to visualize the biofilms of mono- and mixed-species. As shown in Figure 2A, the dynamic development of mono-species biofilm of *V. parahaemolyticus* or *L. monocytogenes* was consistent with the results of CV assay. In essence, the architecture of the biofilm of single strain was denser when compared with that of mixed strains. Mono-species of *V. parahaemolyticus* or *L. monocytogenes* produced additional extensive biofilms structures at the mature stage. In contrast, mixed-species biofilms presented a sparse and dispersed architecture. SEM was used to further verify the morphological structure of mono- and mixed-species biofilms at 36 h. The results showed that cells in the mono-species biofilm adhere to each other to form large aggregates (Figures 3A–D). However, in the mixed-species biofilms, SEM clearly showed the biofilm formation was significantly decreased with less cells and extracellular substance (Figures 3E,F).

Structural parameters were calculated to further characterize the morphological properties of biofilms (Beyenal et al., 2004). Quantitative image analysis revealed that the bio-volume of mono-species biofilms (4.55 × 10^5^ to 6.12 × 10^5^ μm^3^ at 12 h, 1.13 × 10^6^ to 2.68 × 10^6^ μm^3^ within 24–48 h and 1.68 × 10^5^ to 1.21 × 10^6^ μm^3^ within 60–72 h) were higher overall than that of mixed-species biofilms (3.99 × 10^5^ to 4.74 × 10^5^ μm^3^ at 12 h, 5.69 × 10^5^ to 1.10 × 10^6^ μm^3^ within 24–48 h and 1.76 × 10^5^ to 5.76 × 10^5^ μm^3^ within 60–72 h) (Figure 2B), and further supported the results of the CV assay (Figure 1). In addition, we observed similar trends between biofilm mean thickness and biofilm formation (Figures 2B,C), where an increase in the biofilm formation correlates with increase in the biofilm mean thickness. All biofilms were thickest at the maturation stage after 36 h incubation and the biofilm mean thickness of VP802, VP24, LM115, LM5, VP802 + LM115, and VP24 + LM5 were 25.00 ± 2.04, 21.08 ± 2.14, 19.91 ± 2.25, 20.00 ± 1.05, 10.57 ± 2.04, and 9.59 ± 1.57 μm (Figure 2C). In addition, the porosity of a biofilm can also be used as a measure for comparing mono-species biofilm and mixed-species biofilms (Figure 2D). Even though the porosity of all biofilm types was the maximum at 72 h, the porosity of mixed-species biofilms was larger than that of mono-species biofilm at 24–60 h.

### Enumeration of Biofilm and Planktonic Cells

**FIGURE 4:**
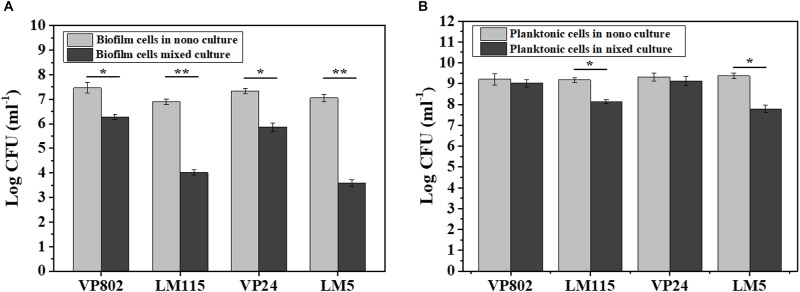
Enumeration of biofilm and planktonic cells in mono-species and mixed-species biofilms by absolute quantitative PCR. **(A)** Biofilm cells in mono- and mixed cultures; **(B)** planktonic cells in mono- and mixed cultures. Error bars represent standard deviation of three biological replicates. ^∗^*p* < 0.05; ^∗∗^*p* < 0.01.

The number of biofilm and planktonic cells of *V. parahaemolyticus* and *L. monocytogenes* in the mono and mixed cultures at the maturation stage were determine by qPCR. Conventional PCR demonstrated that the designed primers were specific for each species and the standard curves of *V. parahaemolyticus* and *L. monocytogenes* were linear (*R*^2^ > 0.99) (Supplementary Figures S1A,B). As shown in Figure 4, *V. parahaemolyticus* had a competitive advantage in the mixed-species culture with a higher population than *L. monocytogenes*. And the number of biofilm and planktonic cells in mixed species were generally lower than that in mono species.

In the mono-species biofilm, the biofilm cells of *L. monocytogenes* LM115 and LM5 were approximately 7 log CFU/mL. In contrast, the biofilm cells of *L. monocytogenes* were only 3–4 log CFU/mL in the mixed-species biofilm (Figure 4A). For the planktonic cells, the number of *L. monocytogenes* in the mono cultures was significantly lower 1–2 log CFU/mL than that in the mixed cultures (*p* < 0.05), while the similar cell population of *V. parahaemolyticus* was observed whether in mono-species or mixed-species cultures (Figure 4B).

### Differences in Gene Transcription in Mono- and Mixed-Species Biofilms

**FIGURE 5:**
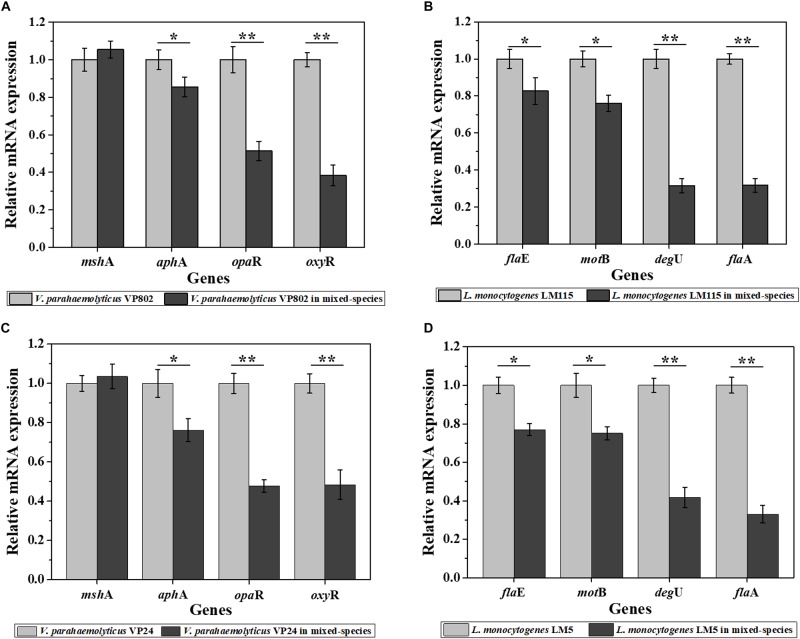
Relative expression levels of genes involved in the regulation of biofilm formation of *V. parahaemolyticus* VP802 **(A)**, *L. monocytogenes* LM115 **(B)**, *V. parahaemolyticus* VP24 **(C)**, and *L. monocytogenes* LM5 **(D)**. The light gray bars represent mono-species biofilm and dark gray bars represent mixed-species biofilms. Error bars represent the standard deviation of triplicate experiments. ^∗^*p* < 0.05; ^∗∗^*p* < 0.01.

As results show in Figure 5, compared with the mono-species biofilms, the biofilm-related genes in the mixed-species biofilms were overall down-regulated. For *V. parahaemolyticus* VP802, biofilm regulatory genes *aph*A, *opa*R, and *oxy*R were downregulate by 1.17, 1.94, and 2.61 fold, respectively, and there was no significant expression changes of *msh*A gene. For *L. monocytogenes* LM115, biofilm regulatory genes *fla*A, *fla*E, *mot*B, and *deg*U were downregulate by 3.14, 1.21, 1.32, and 3.17 fold, respectively (Figures 5A,B). The similar trends in gene down-regulation were observed in another mixed-species group (*V. parahaemolyticus* VP24 and *L. monocytogenes* LM5) (Figures 5C,D).

### Cell Metabolic Activity in Mono- and Mixed-Species Biofilms

**FIGURE 6:**
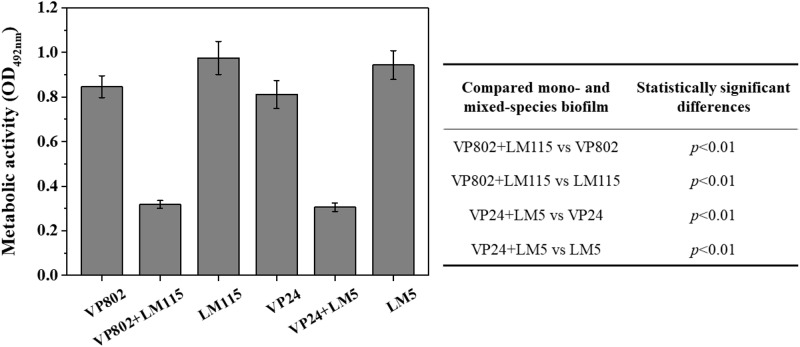
Metabolic activity of mono- and mixed-species biofilms by XTT assay. The table embedded within the figure shows the significance of difference in OD_492 *nm*_ between mono- and mixed-species biofilms. Error bars represent the standard deviation of triplicate experiments.

The cell metabolic activity of the mono- and mixed-species mature biofilms were compared. Results showed that metabolic activity of mixed-species biofilms was significantly weaker than that of mono-species biofilm (Figure 6, *p* < 0.01). In mono-species biofilm, the OD_492 *nm*_ of *V. parahaemolyticus* VP802, *V. parahaemolyticus* VP24, *L. monocytogenes* LM115 and *L. monocytogenes* LM5 were 0.85 ± 0.04, 0.81 ± 0.06, 0.97 ± 0.07, and 0.94 ± 0.06, respectively. In mixed-species biofilms, the OD_492 *nm*_ of VP802 + LM115 and VP24 + LM5 were only 0.32 ± 0.02 and 0.30 ± 0.02, respectively. That indicated the cell viability will decrease when *V. parahaemolyticus* and *L. monocytogenes* co-cultured to form a mixed biofilm.

### Spatial Distribution of *V. parahaemolyticus* and *L. monocytogenes* in Mixed-Species Biofilm

**FIGURE 7:**
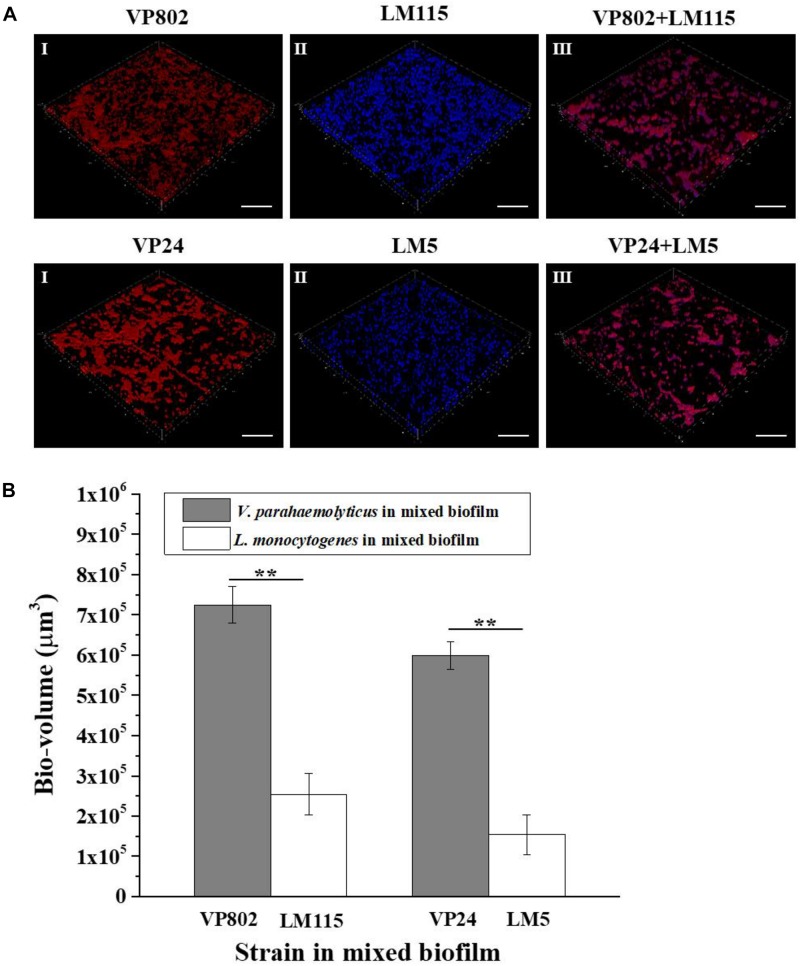
Spatial organization of mixed-species biofilms using FISH-CLSM. **(A)** Images (captured under the 63õ oil-immersion objective) showing the mono- and mixed-species biofilms of *V. parahaemolyticus* (Red) and *L. monocytogenes* (Blue) after 36 h cultivation. Scale bar: 20 μm. **(B)** Bio-volumes of each species in mixed biofilm were obtained by pixel analysis based on images. Error bars represent standard deviation of three biological replicates. **I**: *V. parahaemolyticus*; **II**: *L. monocytogenes*; **III**: Mixed-species. ^∗∗^*p* < 0.01.

Fluorescence *in situ* hybridization in combination with CLSM was used to visualize the spatial distribution the two pathogenic strains in the mixed biofilm at the maturation stage (36 h). The probes used for FISH showed good specificity for the strains as no abnormal signal was detected in the mono-species biofilm (Figures 7AI,II). Most of the *L. monocytogenes* (Blue) cells were located at the bottom of the mixed biofilm and enclosed by *V. parahaemolyticus* (Red) (Figure 7AIII). The microbial community of *V. parahaemolyticus* or *L. monocytogenes* in a mono species biofilm formed much denser biofilm compared to mixed-species biofilms and showed high concordance with the results of CLSM (Figure 2A). For confirmation, we quantified the pixels of *V. parahaemolyticus* or *L. monocytogenes* in each layer of the FISH-CLSM images and showed that the bio-volume of *V. parahaemolyticus* in the mixed biofilm was much higher than that of *L. monocytogenes* (Figure 7B). The ratios of bio-volume of VP802 to LM115 and VP24 to LM5 were 2.9:1 and 3.6:1, respectively.

### Chemical Variation in EPS of Biofilm

**FIGURE 8:**
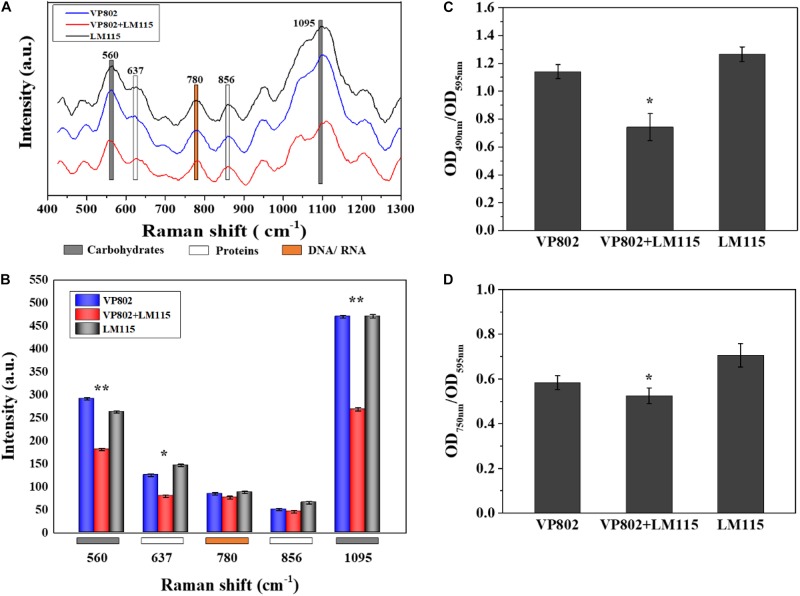
Chemical variation in EPS between mono- and mixed- species biofilm. **(A)** Raman spectrum of EPS of mono- and mixed-species biofilms formed by *V. parahaemolyticus* VP802 and *L. monocytogenes* LM115. **(B)** Intensities changes. Error bars indicated the standard deviations of six measurements. ^∗^*p* < 0.05; ^∗∗^*p* < 0.01. **(C)** Extracellular polysaccharide (OD_490 *nm*_/OD_595 *nm*_) and **(D)** Extracellular protein (OD_750 *nm*_/OD_595 *nm*_) in EPS of mono- and mixed-species biofilms formed by *V. parahaemolyticus* VP802 and *L. monocytogenes* LM115. Error bars indicated the standard deviations of three measurements. ^∗^*p* < 0.05; ^∗∗^*p* < 0.01.

The EPS of mono- and mixed-species biofilms was extracted to investigate its chemical and structural variation. Raman spectra of the EPS in the spectral range of 425–1300 cm^–1^ are shown in Figure 8A. The dominant peaks and their assignments are summarized in Table 3 and these data are related to previous studies (Ivleva et al., 2009; Ramya et al., 2010; Chao and Zhang, 2012; Samek et al., 2014; Kusic et al., 2015). The major peaks at 560, 637, 780, 856, and 1095 cm^–1^ can be assigned as carbohydrates, proteins and nucleic acids. In detail, peaks of 560, 1090–1095 cm^–1^ were considered as carbohydrates, because 560, 1090–1095 cm^–1^ were assigned to C-O-C glycosidic ring deformation vibration, C-C stretching and C-O-C glycosidic link; peaks at 637 and 856 cm^–1^ corresponded to the proteins, because 637 and 856 cm^–1^ were assigned to C-S stretching and C-C twisting proteins (tyrosine); the peak at 780 cm^–1^ was considered as the typical peak of nucleic acids owing to the cytosine and uracil ring stretching modes at 780 cm^–1^.

**TABLE 3:** Assignment of the Raman bands of biofilm matrix.

**Raman shift (cm^–1^)**	**Assignment**	**Macromolecular assignment**	**References**
560–582	C-O-C glycosidic ring def polysaccharide; COO- wag; C-C skeletal	Carbohydrates	Chao and Zhang, 2012; Kusic et al., 2015; Tan et al., 2018
637–695	C–S str and C–C twisting of proteins (tyrosine)	Proteins	Ivleva et al., 2009; Ramya et al., 2010; Chao and Zhang, 2012
780–785	C,U	DNA/RNA	Ivleva et al., 2009; Samek et al., 2014
854–856	Ring breath, tyr	Proteins	Kusic et al., 2015
1,090–1,095	C-C str, C-O-C glycosidic link; ring br, sym	Carbohydrates	Ivleva et al., 2009; Tan et al., 2018

**def*, deformation vibration; *str*, stretching; *try*, tyrosine; *br*, breathing; *sym*, symmetric.*

Compared with the mono-species biofilms, peaks intensity of 560, 637, and 1095 cm^–1^ significantly (*p* < 0.05) decreased in the mixed biofilm (Figure 8B), suggesting that the intensity of carbohydrates and proteins in the EPS were significantly reduced when *V. parahaemolyticus* and *L. monocytogenes* were co-cultured. However, there was no obvious difference in the peak of 780 cm^–1^, implying that the co-cultivation of *V. parahaemolyticus* and *L. monocytogenes* didn’t obviously change nucleic acid components.

The amounts of proteins and polysaccharides in EPS were also quantified by the phenol-sulfuric acid method and Lowry method. As shown in Figures 8C,D, the amount of carbohydrates and proteins in the EPS of mixed-species biofilms were lower than that in mono-species biofilm and these results were consistent with the Raman spectrum assay (Figure 8B). In addition, the percentage decrease in polysaccharide was higher than that of protein, indicating that extracellular polysaccharide is the main contributor to the structural reduction of mixed biofilm.

### Impact of Mixed-Species Community on Their Susceptibility to Antibiotics

Two commonly used antibiotics (ciprofloxacin and cefotaxime sodium) were used to explore the effects of interactions between the two-bacterial species on their antibiotic susceptibility. Results of MBIC for each biofilm are depicted in Table 4, where the MBIC of ciprofloxacin to VP802, VP802 + LM115, and LM115 in biofilms were 2, 1, 2 μg/mL. The MBIC of Cefotaxime sodium to VP802, VP802 + LM115, and VP24 in biofilms was 32, 16, and 128 μg/mL, respectively. In general, *V. parahaemolyticus* and *L. monocytogenes* in mono-species biofilm presented higher MBIC values to ciprofloxacin and cefotaxime sodium than those in mixed species biofilm.

**TABLE 4:** Antibiotic susceptibility of bacteria in biofilms.

**Biofilm**	**MBIC (μ g/mL)**	
	**CIP**	**CTX**
Mono-species biofilm of VP802	2	32
Mixed-species biofilm of VP802 and LM115	1	16
Mono-species biofilm of LM115	2	128

*MBIC, minimum biofilm inhibitory concentration; CIP, ciprofloxacin; CTX, cefotaxime sodium.*

## Discussion

Biofilm formation is an effective strategy for bacteria to survive harsh environments. These environments include dental plaques, soil, wastewater pipelines, medical devices and food-processing surfaces (Filoche et al., 2004; Moons et al., 2009; Elias and Banin, 2012; Moretro and Langsrud, 2017; Si and Quan, 2017). Biofilms in nature are structured communities composed of more than one species and the diversity of these species and their interspecies interactions results in complex biofilms which exhibit a variety of structural and functional characteristics (Nadell et al., 2009; Yang et al., 2011). For instance, the resistance of *L. monocytogenes* to peracetic acid is enhanced in a mixed-species biofilm with *Lactobacillus plantarum*, while *E. coli* O157:H7 and *Salmonella* are more sensitive to levulinic acid plus SDS in mixed-species biofilms (van der Veen and Abee, 2011; Chen et al., 2015). Therefore, understanding biofilm development of mixed-species increases the chances of developing novel tools for removing these biofilms.

We developed mono- and mixed-species biofilms consisting of *V. parahaemolyticus* and *L. monocytogenes* in this study and have characterized the complexities in these biofilms. Results from CV and CLSM images confirmed three distinct development stages: adhesion and growth stage, biofilm maturity stage and biofilm collapse stage (Hall-Stoodley et al., 2004). At the maturity stage, single species biofilm of *V. parahaemolyticus* and *L. monocytogenes* exhibited firm and dense matrices, while these properties was significantly reduced in mixed-species biofilms. And SEM provided the further evidence to confirm the observation of CLSM images (Figure 3). The weakened biofilm structure of this mixed species biofilm is possibly the result of microbial competition for nutrients and inhibition of the growth of the other co-existing species (Yang et al., 2011).

Quantitative analysis of CSLM images provides an additional tool for characterizing biofilm structures (Yang et al., 2000; Beyenal et al., 2004). Mature mixed biofilms were thinner and their biological volume was also lower when assessed using quantitative CSLM in comparison to mono-species biofilms (Figures 2B,C). Co-culturing of *V. parahaemolyticus* and *L. monocytogenes* was therefore detrimental to biofilm formation. It has been reported that an increase in biofilm thickness can also be caused by bacteria been unable to be detached from the mature biofilm (Vuong et al., 2004). In such cases, porosity which is defined as the ratio of void area to total area (Yang et al., 2000) can be used to evaluate the structural integrity of the biofilm. Porosity of the mixed-species biofilms was larger when compared to a mono species biofilm, leading to sparse structures in the biofilm and significant structural collapse after 72 h incubation (Figure 2D). These additional observations confirmed that co-culture of *V. parahaemolyticus* and *L. monocytogenes* reduces adhesion ability of the formed biofilm.

A number of studies have found that *L. monocytogenes* are inhibited in biofilms when other bacteria are present (Leriche and Carpentier, 2000; Norwood and Gilmour, 2000; Carpentier and Chassaing, 2004). Therefore, we hypothesized that the presence of *V. parahaemolyticus* would interfere with *L. monocytogenes* adhering to the contact surface, which is the initial step in biofilm formation. Obtained results showed that the density of *V. parahaemolyticus* was higher than that of *L. monocytogenes* in mixed-species biofilms. And both biofilm and planktonic cells of *L. monocytogenes* were significantly reduced in mixed-species cultures compared with mono ones (Figure 4). That concluded that there was a competitive relationship between these two pathogens, and *V. parahaemolyticus* had a competitive advantage in the mixed-species biofilms.

In addition, the differences in transcription and metabolism of *V. parahaemolyticus* or *L. monocytogenes* were further analyzed to explore the causes of biofilm reduction in the mixed-species biofilms. For *V. parahaemolyticus*, *aph*A and *opa*R are the master regulator genes of quorum sensing system, which are critical for biofilm formation (Zhang et al., 2016). *oxy*R and *msh*A are the flagellar and IV pili genes, respectively, contributed to bacterial adhesion (Shime-Hattori et al., 2006; Chung et al., 2016). For *L. monocytogenes*, *deg*U, *fla*A, *fla*E and *mot*B are flagellar genes, which play an important role in controlling bacterial adhesion to regulate biofilm formation (Knudsen et al., 2004; Gueriri et al., 2008; Kumar et al., 2009). Compared with the mono-species biofilms, these biofilm-related genes in the mixed-species biofilms were down-regulated, which might be an important cause for the reduction of mixed-species biofilms (Figure 5). Besides, XTT assay demonstrated that the metabolic activity of the mixed-species biofilm cells was obviously weaker than that in the mono-species biofilm (Figure 6). According to description previously, the cell metabolic activity is closely related to the formation and structure of biofilms (Hoiby et al., 2010). The weaker metabolic activity could be a favorable evidence to explain the reduction of mixed-species biofilms.

Structural characterization and cell localization in biofilms are essential for understanding the development process of mixed-species biofilm (Liu et al., 2018). It was reported that bacteria may alter their biological traits to adapt to the local environment and this adaptation is reflected in the spatial orientation of species in a structured community (Hansen et al., 2017; Liu et al., 2018). Phalak et al. (2016) found that *P. aeruginosa* accumulated at the top of the biofilm and inhibited the oxygenation of *S. aureus* at the bottom of the biofilm. On this basis, FISH-CLSM was conducted to clarify the spatial localization of *V. parahaemolyticus* and *L. monocytogenes* in a mature mixed biofilm. From the three-dimensional images, most of *V. parahaemolyticus* were located at the surface layers of the mixed biofilm, while *L. monocytogenes* were distributed at the bottom of the mixed biofilm (Figure 7). This spatial orientation of *V. parahaemolyticus* therefore improves the competition for resources.

Research has shown that the chemical composition of biofilms varied greatly from mono-species to dual species (Ramirez-Mora et al., 2018). The adhesion ability of a bacterial biofilm is closely related to EPS (Tan et al., 2018) and components of EPS are responsible for biofilm properties, such as density, porosity, and hydrophobicity (Liu et al., 2007; Yang et al., 2011). Destruction or dissolution of EPS can allow disinfectant to successfully target cells in the biofilm. Therefore understanding the changes in the chemical composition of EPS of mixed biofilms improves the chances for exploiting weaknesses in similar pathogenic biofilms (Shen et al., 2016). In this study, we found that carbohydrates were the most abundant carbon compound in EPS of mono-species biofilm, followed by protein and nucleic acid. These compounds were significantly reduced in mixed-species biofilms (*p* < 0.05) except or nucleic acid (Figure 8). The reduction of polysaccharide and proteins might be the important factor causing the collapse of mixed-species biofilms. Indeed, the molecular mechanism of reduction in mixed-species biofilms should be further explored by transcriptome, proteome, or metabolomics analysis.

Antibiotics may be prevented from penetrating a biofilm as the EPS matrix acts as a physical barrier and a well-structured biofilm increases resistance to antibiotic treatments (Meyer, 2003; Olsen, 2015). A reduction of EPS in a mixed- *V. parahaemolyticus* and *L. monocytogenes* species biofilms would therefore increase the exposure of these pathogens to disinfectants (Shen et al., 2016). In this study, the BMIC test showed that the susceptibility of bacterial cells to ciprofloxacin and cefotaxime sodium in mixed-species biofilms was generally increased, probably due to the reduction of EPS, which made the structure of mixed-species biofilm weaker than mono ones. Therefore, the presence of competitive interactions between *V. parahaemolyticus* and *L. monocytogenes* ultimately increases the susceptibility of mixed-species biofilms to antibiotics.

## Conclusion

Mono- and mixed-species biofilms formed by *V. parahaemolyticus* and *L. monocytogenes* was first compared in this study. The biomass, bio-volume, and thickness of mixed-species biofilm was much less than that of each individual mono-species biofilm. And these variations might result from the reduction of bacterial numbers, biofilm-regulated genes expression, metabolic activity, extracellular polysaccharide and protein in mixed-species biofilm. In addition, both *V. parahaemolyticus* and *L. monocytogenes* were more susceptible to antibiotics when grown together in a mixed-species biofilm. This study contributes to the mechanistic understanding of interactions in mixed species biofilm formation and increases the chance of developing innovative sanitizers for removing these types of biofilms.

## Data Availability Statement

All datasets generated for this study are included in the article/Supplementary Material.

## Author Contributions

YZ, YP, and HL conceived and supervised the study. PC and JY designed the experiments. PC performed the experiments and wrote the manuscript. PC, BH, LT, and JW analyzed the data. JW, ZZ, and YZ revised the manuscript.

## Conflict of Interest

The authors declare that the research was conducted in the absence of any commercial or financial relationships that could be construed as a potential conflict of interest.
