# Supplementary material for: Characterization of Mixed-Species Biofilm Formed by Vibrio parahaemolyticus and Listeria monocytogenes
Source: Front Microbiol. 2019 Nov 8;10:2543. doi: 10.3389/fmicb.2019.02543 (PMC6856058; doi:10.3389/fmicb.2019.02543)
Supplement: Supplementary file 1 [file Data_Sheet_1.pdf]

Characterization of mixed-species biofilm formed by *Vibrio parahaemolyticus* and *Listeria monocytogenes*

Ping Chen[1], Jingjing Wang[1,2,3], Bin Hong[1], Ling Tan[1], Jun Yan[1,2,3], Zhaohuan Zhang[1], Haiquan Liu[1,2,3,4], Yingjie Pan[1,2,3], Yong Zhao[1,2,3*]

[1] College of Food Science and Technology, Shanghai Ocean University, Shanghai 201306, China;
[2] Shanghai Engineering Research Center of Aquatic Product Processing & Preservation, Shanghai 201306, China;
[3] Laboratory of Quality & Safety Risk Assessment for Aquatic Products on Storage and Preservation (Shanghai), Ministry of Agriculture, Shanghai 201306, China;
[4] Engineering Research Center of Food Thermal-processing Technology, Shanghai Ocean University, Shanghai 201306, China;

Corresponding author: Tel/fax: +86 21 6190 0503; E-mail: yzhao@shou.edu.cn (Yong Zhao).

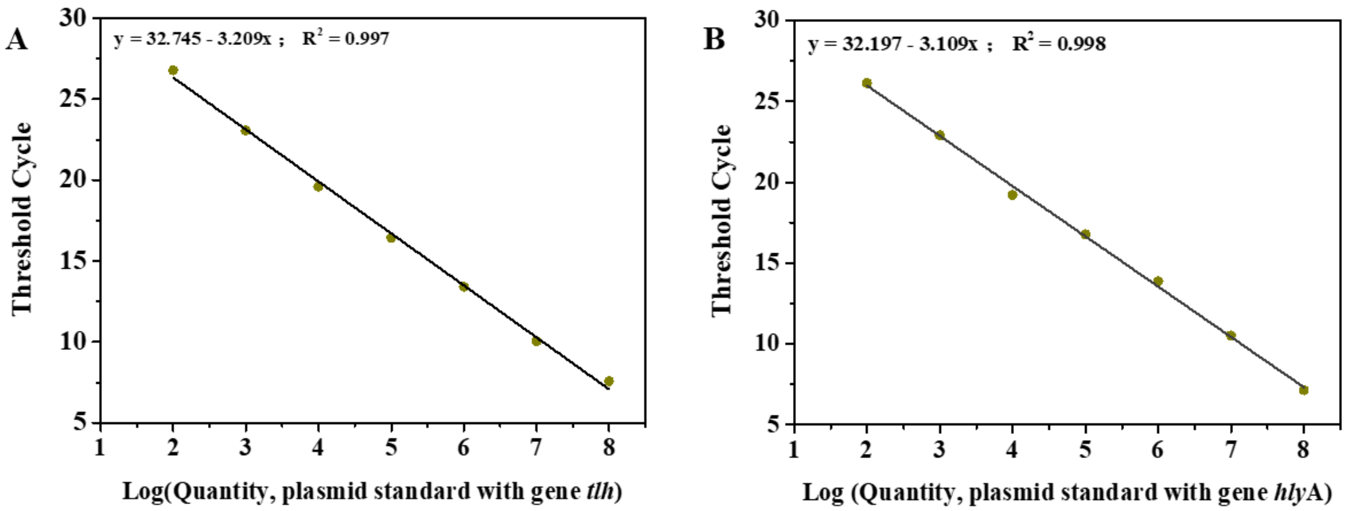

FIGURE S1: Standard curves for real-time qPCR in pure culture. A: Standard curves for *V. parahaemolyticus* based on plasmid standard with *tlh*. **B**: Standard curves for *L. monocytogenes* based on plasmid standard with *hly*A.

Table S1 | Primer sequences for the RT-qPCR assay

Target gene	Nucleotide sequence (5'→3')	Reference
*rec*A	F - GCTAGTAGAAAAAGCGGGTG<br>R - ATACGAGTGGTTGCTGTCATG	Ma et al., 2015
*msh*A	F - GGTTTCGTTTAGGTCACG<br>R - CGTCGAAATGTCGGCGG	Shime-Hattori et al., 2006
*aph*A	F – ACACCCAACCGTTCGTGATG<br>R - GTTGAAGGCGTTGCGTAGTAAG	Wang et al., 2013
*opa*R	F - TGTCTACCAACCGCACTAACC<br>R - GCTCTTTCAACTCGGCTTCAC	Zhang et al., 2016
*oxy*R	F - TCGTCAGCTAGAGGAAGG<br>R - TGGTCGCGTAAGCAATGC	Chung et al., 2016
Gap	F-AAAGCTGGCGCTAAAAAAGTTG<br>R-TTCATGGTTTACATTGTAAACGATTG	Mattila et al., 2011
*fla*E	F - CAGCAGGTTCCCCGACTTC<br>R - CGGCCTTGTAGTGCTGCAT	This study
*mot*B	F - TGCAAAAAAATTCGAACAAATGG<br>R - CTGCCGCGCCTTCCT	This study
*deg*U	F - GGAGGAGTAGTCATTATGGC<br>R - ACTTCTGGTTGTTGGTAGCC	This study
*fla*A	F - GAAGGCATGACTCAAGCGCA<br>R - GCAAGACCAGCAGCGTCATC	This study

REFRENCES

Chung, C.H., Fen, S.Y., Yu, S.C., and Wong, H.C. (2016). Influence of oxyR on Growth, Biofilm Formation, and Mobility of *Vibrio parahaemolyticus*. Applied and Environmental Microbiology 82(3), 788-796. doi: 10.1128/AEM.02818-15

Ma, Y.J., Sun, X.H., Xu, X.Y., Zhao, Y., Pan, Y.J., Hwang, C.A., et al. (2015). Investigation of Reference Genes in *Vibrio parahaemolyticus* for Gene Expression Analysis Using Quantitative RT-PCR. Plos One 10(12), e0144362. doi: 10.1371/journal.pone.0144362

Mattila, M., Lindström, M., Somervuo, P., Markkula, A., and Korkeala, H. (2011). Role of flhA and motA in growth of *Listeria monocytogenes* at low temperatures. International Journal of Food Microbiology 148(3), 177-183. doi: 10.1016/j.ijfoodmicro.2011.05.022

Shime-Hattori, A., Iida, T., Arita, M., Park, K.S., Kodama, T., and Honda, T. (2006). Two type IV pili of *Vibrio parahaemolyticus* play different roles in biofilm formation. Fems Microbiology Letters 264(1), 89-97. doi: 10.1111/j.1574-6968.2006.00438.x

Wang, L., Ling, Y., Jiang, H.W., Qiu, Y.F., Qiu, J.F., Chen, H.P., et al. (2013). AphA is required for biofilm formation, motility, and virulence in pandemic *Vibrio parahaemolyticus*. International Journal of Food Microbiology 160(3), 245-251. doi: 10.1016/j.ijfoodmicro.2012.11.004

Zhang, Y.Q., Zhang, L.Y., Hou, S.N., Huang, X.X., Sun, F.J., and Gao, H. (2016). The Master Quorum-Sensing Regulator OpaR is Activated Indirectly by H-NS in *Vibrio parahaemolyticus*. Current Microbiology 73(1), 71-76. doi: 10.1007/s00284-016-1018-8
